# Quantifying electron cascade size in various irradiated materials for free-electron laser applications

**DOI:** 10.1107/S1600577522000339

**Published:** 2022-02-15

**Authors:** Vladimir Lipp, Igor Milov, Nikita Medvedev

**Affiliations:** a Institute of Nuclear Physics, Polish Academy of Sciences, Radzikowskiego 152, 31-342 Kraków, Poland; b Center for Free-Electron Laser Science CFEL, Deutsches Elektronen-Synchrotron DESY, Notkestrasse 85, 22607 Hamburg, Germany; c Industrial Focus Group XUV Optics, MESA+ Institute for Nanotechnology, University of Twente, Drienerlolaan 5, 7522 NB Enschede, The Netherlands; d Institute of Physics, Czech Academy of Sciences, Na Slovance 2, 18221 Prague 8, Czech Republic; e Institute of Plasma Physics, Czech Academy of Sciences, Za Slovankou 3, 18200 Prague 8, Czech Republic

**Keywords:** electron cascades, X-ray free-electron lasers, Monte Carlo, photon-induced cascade, electron transport

## Abstract

Studying electron- and X-ray-induced electron cascades in solids is essential for various research areas at free-electron laser facilities, such as X-ray imaging, crystallography, pulse diagnostics or X-ray-induced damage. To better understand the fundamental factors that define the duration and spatial size of such cascades, this work investigates the electron propagation in ten solids relevant for the applications of X-ray lasers: Au, B_4_C, diamond, Ni, polystyrene, Ru, Si, SiC, Si_3_N_4_ and W. Using classical Monte Carlo simulation in the atomic approximation, we study the dependence of the cascade size on the incident electron or photon energy and on the target parameters. The results show that an electron-induced cascade is systematically larger than a photon-induced cascade. Moreover, in contrast with the common assumption, the maximal cascade size does not necessarily coincide with the electron range. It was found that the cascade size can be controlled by careful selection of the photon energy for a particular material. Photon energy, just above an ionization potential, can essentially split the absorbed energy between two electrons (photo- and Auger), reducing their initial energy and thus shrinking the cascade size. This analysis suggests a way of tailoring the electron cascades for applications requiring either small cascades with a high density of excited electrons or large-spread cascades with lower electron densities.

## Abstract 2

Studying electron- and X-ray-induced electron cascades in solids is essential for various research areas at free-electron laser facilities, such as X-ray imaging, crystallography, pulse diagnostics or X-ray-induced damage. To better understand the fundamental factors that define the duration and spatial size of such cascades, this work investigates the electron propagation in ten solids relevant for the applications of X-ray lasers. Using classical Monte Carlo simulation in the atomic approximation, the dependence of the cascade size on the incident electron or photon energy and on the target parameters is studied.

## 1. Introduction

Femtosecond X-ray lasers have opened up new frontiers in physics, driving a wide variety of applications such as ultrafast crystallography (Patterson, 2014 ▸), single-particle imaging using Coulomb explosion (Ablikim *et al.*, 2016 ▸) or X-ray-induced photo-electrons (Kastirke *et al.*, 2020 ▸), material processing and nanostructuring (Dinh *et al.*, 2019 ▸), catalysis (Bergmann *et al.*, 2021 ▸), biophysics (Thor & Madsen, 2015 ▸), and biomedicine (Melissinaki *et al.*, 2011 ▸). Many applications depend on the parameters of the electron cascades induced by X-ray photon absorption.

For instance, in the case of diffract-and-destroy experiments (Chapman *et al.*, 2011 ▸; Spence, 2017 ▸), electron cascades are an unwanted side effect, which can significantly complicate the measurement by deteriorating the diffraction patterns (Nass, 2019 ▸). Reducing the damage caused by the electrons is a key issue in this field (Spence, 2017 ▸). One way to achieve this would be to spread the electron cascade in space, thereby reducing the density of excited electrons and energy density they carry. For optical elements and mirrors at free-electron lasers (FELs) (Gaudin *et al.*, 2009 ▸; Koyama *et al.*, 2016 ▸; Milov *et al.*, 2020 ▸), it is also crucial to dissipate electron energy over the largest volume possible to reduce the damage caused by the photoabsorption and subsequent electron cascading.

In the application to the FEL pulse shape monitor (Chalupský *et al.*, 2010 ▸; Pikuz *et al.*, 2015 ▸), the opposite is true: the cascades are required to be localized, to create a sufficient electron density directly where the FEL pulse impinges the surface. When the spread of electrons is too wide, the signal is smeared out, which hinders the determination of the laser pulse spatial shape. Monitoring the FEL pulse duration and its temporal profile via cross-correlation with an optical probe (Harmand *et al.*, 2013 ▸; Riedel *et al.*, 2013 ▸) also requires cascades to be localized quickly (Medvedev, 2015 ▸).

The targets studied in this work are relevant for X-ray applications. For instance, Ru and B_4_C are used in X-ray mirrors (Milov *et al.*, 2018 ▸; Aquila *et al.*, 2015 ▸), W and B_4_C can be used as parts of the multilayer components in X-ray optics (Bajt *et al.*, 2018 ▸; Follath *et al.*, 2019 ▸), and Si_3_N_4_ is widely used for FEL-pulse diagnostics (Tkachenko *et al.*, 2021 ▸).

It is thus essential to find reliable ways to control electron cascades for different applications. One of the common assumptions about the cascade size is that it is defined by the electron range (*e.g.* Pikuz *et al.*, 2015 ▸). We test the limits of this assumption and demonstrate cases when it does not hold. We discuss the underlying physics of electron cascade development and suggest how this knowledge can be utilized for practical needs. We then propose a simple scheme for manipulating the cascade size via tailoring either the material or the photon energy.

## 2. Model

We applied the *XCascade3D* code (Medvedev, 2015 ▸; Lipp *et al.*, 2017 ▸), which is based on the classical asymptotic trajectory Monte Carlo (MC) simulation method. It models the following processes: photoabsorption, which excites electrons from core or valence atomic shells of the target; photo-electron propagation accompanied by elastic (change of direction) and inelastic (impact ionization) scattering events; Auger decays of core holes, resulting in emission of secondary electrons; and transport of all the secondary-generation electrons. Fluorescent decay of core holes is neglected since the Auger decay channel dominates for all the materials and energies considered in this work. No approximation of condensed history is used throughout the code, and all processes of all particles are modelled event-by-event (Salvat & Fern, 2015 ▸).

The target is assumed to be a homogeneous arrangement of atoms with a solid density. All core and valence shells of elements are considered to have ionization potentials of individual atoms, neglecting effects of the band structure, which is a standard approximation in Monte Carlo modelling (Jacoboni & Reggiani, 1983 ▸; Jenkins *et al.*, 1988 ▸; Salvat & Fern, 2015 ▸). Ionization potentials for all elements are taken from the EPICS2017 database (EADL part) (Cullen, 2018 ▸). The effects of the density of states are neglected, and valence electronic energy levels are treated as atomic levels. This approximation does not significantly affect electronic transport at energies above 50 eV. Since electrons with <50 eV only perform a few inelastic collisions and quickly lose their energy below a cut off (equal here to 10 eV), their transport does not noticeably affect the overall simulation results. Thus, this atomic approximation of the target should be satisfactory for the purposes of this work.

Photoabsorption cross sections are also taken from the EPICS2017 database (EPDL part) (Cullen, 2018 ▸). For simplicity and without affecting presented results, we assume linear beam polarization and that each photo-electron is emitted in one of the two directions along the polarization axis. The energy of such an electron is defined by the difference between the photon energy and the ionization potential of the shell it is emitted from. The shell absorbing the photon is sampled according to the partial photo-absorption cross sections among all the atomic shells of all chemical elements of the target (Medvedev, 2015 ▸). For compound targets, the total cross section is calculated from the atomic ones of all elements using the Bragg additivity rule (Salvat & Fern, 2015 ▸).

Core holes created by the photoabsorption (as well as by electrons via impact ionization) relax via Auger decay with characteristic times taken from the EPICS2017 database (Cullen, 2018 ▸) [except for the 1*s* shell of boron and 5*p* shell of tungsten, for which the Auger decay times are taken from the work by Keski-Rahkonen & Krause (1974 ▸) as 9.7 fs and 0.05 fs, respectively]. The shells participating in the Auger decay are randomly chosen according to the probabilities from EPICS2017. In such decay, the core hole disappears, and two new holes in the upper-lying shells of the same atom appear, and an electron is emitted with the energy defined by the ionization potentials of the shells involved: *E*_Au_ = *I*_p_(core) − *I*_p_(1) − *I*_p_(2), where *E*_Au_ is the kinetic energy of the Auger electron, *I*_p_(core) is the ionization potential of the decaying core hole and *I*_p_(1,2) are the ionization potentials of the first and second shells participating in the Auger decay. Auger cascades are traced until all holes end up in the valence shells that cannot decay within the atomic approximation.

In molecules, and by extension in solids, inter-atomic Auger decay is also possible, in which electrons from neighbouring atoms are involved (Medvedev *et al.*, 2010 ▸). This mechanism is generally subdominant to intra-atomic Auger, except for a number of specific situations which do not occur under the conditions studied. Thus, no inter-atomic Auger decays are taken into account in the current implementation.

Auger, photo- and secondary electrons travel in straight lines until their next collision. They change their direction of motion only in a scattering event, which is assumed to be instantaneous. Coulombic attraction between electrons and holes or repulsion between excited electrons is not included in the model, based on the assumption that the density of excited electrons and holes should be sufficiently low (below ∼10% of atomic density) not to affect their transport significantly. The dominant scattering channel of excited electrons is on the target atoms, and not among themselves (Medvedev *et al.*, 2015 ▸). The electron mean-free path is defined by the total scattering cross section, and sampled according to the Poisson distribution (Jenkins *et al.*, 1988 ▸; Salvat & Fern, 2015 ▸).

Inelastic scattering of electrons – impact ionization – is simulated with the help of the binary-encounter-Bethe (BEB) cross sections (Kim & Rudd, 1994 ▸). This is an atomic approximation to scattering cross sections that neglects the collective effects of a solid (such as plasmon creation and band structure). The parameters, entering the cross-section calculations (an ionization potential, kinetic energy of a shell), are taken from EPICS2017 (Cullen, 2018 ▸). The energy transferred to the excited electron is sampled according to the differential BEB cross section (Medvedev, 2015 ▸). Its kinetic energy is the difference between the transferred energy and the ionization potential of the shell it is excited from. The momentum of the electron and the change of momentum of the incident electron are calculated according to energy and momentum conservation prior to subtracting the ionization potential.

Elastic scattering in our model assumes a change of the incident electron propagation direction only, without changing its energy. Atomic recoil is neglected, as we are not tracing the heating of the atomic system. Although atomic heating may be important for some effects and applications, it typically takes place over longer (picosecond) timescales. At femtosecond timescales and low fluences modelled here, this effect is typically negligible (Medvedev *et al.*, 2015 ▸). Since we are only interested here in the electron cascade size, and are not modelling further atomic responce to heating, it is neglected in the present model. We use Mott’s cross section with the modified Molier screening parameter for elastic scattering (Jenkins *et al.*, 1988 ▸). Jenkins *et al.* (1988 ▸) demonstrated that this approximation reproduces the atomic elastic scattering cross section very well across a wide range of electronic energies. A deflection angle is sampled according to the approximate universal formula from the literature [see Okhrimovskyy *et al.*, 2002 ▸ and equation (7) therein].

All calculated results are averaged over many MC iterations. We perform 10^4^–10^5^ MC iterations, depending on the incident particle energy, to gather reliable statistics. All details of the implementation of the *XCascade3D* code can be found in the literature (Medvedev, 2015 ▸; Lipp *et al.*, 2017 ▸). In the next section, we validate the quantitative precision of the *XCascade3D* model by comparing the calculated electron ranges with the existing data. The model construction described enables very efficient, simple and reliable simulations in the atomic approximation of a wide range of target materials, including light and heavy monoatomic materials as well as compounds of any complexity.

## 3. Results

### 3.1. Definitions

We simulate electron cascades induced by either X-ray photons or electrons. We trace all the electrons, primary and secondary, until they lose their energy below a predefined cut-off, which is typically set equal to 10 eV (Thompson *et al.*, 2009 ▸). The coordinates where this occurs are called the stopping point of an electron.

Although electrons do not simply stop at any energy cut off, it makes for a convenient (and standard) definition, which allows us to compare various models and estimate the distance of a ballistic electron transport, until the electron loses the majority of its energy and the transport turns diffusive (Apostolova *et al.*, 2021 ▸). We avoid the common continuous slowing-down approximation (Thompson *et al.*, 2009 ▸), and instead calculate the incident electron ‘range’ as the distance between the point of its impact and its stopping point (averaged over all MC iterations). Analogously, the photo-electron range is the distance between the point where the photo-electron was created and its stopping point. In the same way, we define the range of Auger electrons.

Additionally, we define the cascade ‘size’ as the distance between the starting point of the cascade and the farthest stopping point among all electrons created in the cascade. The starting point is either the electron incidence point (for electron impact) or the photo-electron creation point (for photon impact).

We calculate the cascade duration by evaluating how the time derivative of the total electron density evolves over time [for details see Medvedev (2015 ▸)]. It is equal to the full width at half-maximum (FWHM) of the derivative of the density. Alternatively, it may also be estimated as the time when the electron density reaches 90% of its maximum, which we also show for a more comprehensive picture.

As we will see below, since an electron scatters and changes its direction during flight, its average range is typically shorter than the maximal size of the cascade. Some secondary electrons, on average, may be found beyond the range of the incident electron. This is the physical mechanism behind the difference reported in the next sections.

### 3.2. Model validation

**Figure 1:**
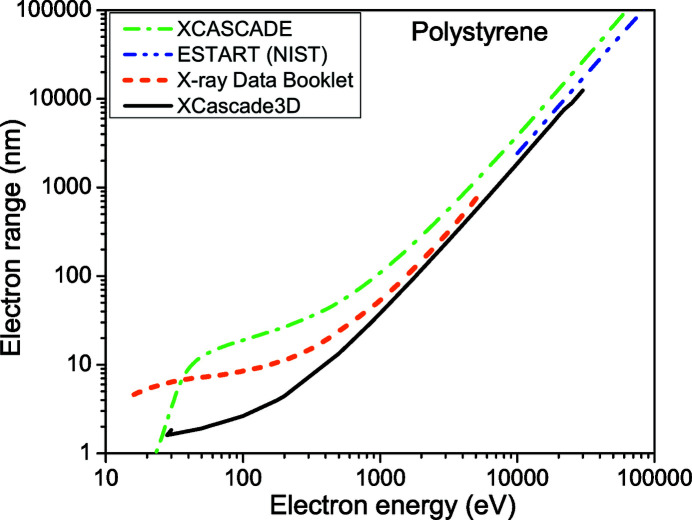
Electron range in polystyrene according to *XCascade3D* simulations – solid black line; *XCASCADE* estimation within the continuous-slowing-down approximation – green dash-dotted line; energy loss function integral from the NIST database – blue dash-dot-dotted line (Berger *et al.*, 1998 ▸); X-ray Data Booklet – orange short-dashed line (Thompson *et al.*, 2009 ▸).

We start by comparing the electron ranges obtained with the *XCascade3D* code defined above with known data, thus verifying its quantitative precision, see Figs. 1 ▸ ▸–3 ▸. Note that in the work by Lipp *et al.* (2017 ▸), the curves calculated with *XCascade3D* differ from those presented here due to a bug fix in the core *XCASCADE* code (Medvedev, 2019 ▸).

As expected, due to the atomic approximation, *XCascade3D* provides very good agreement with other models at high electronic energy. For lower energy, the agreement worsens but stays reasonable. This result is expected as available cross sections differ at low energies. Also, the definition of the electron range in *TREKIS* (Medvedev *et al.*, 2015 ▸) includes only inelastic scattering, which seems to be the reason for a larger discrepancy at low energies.

We conclude that the simulation tool has a reasonable quantitative precision and should provide new insights into the electron dynamics induced by X-ray lasers. In previous works (Mecseki *et al.*, 2018 ▸; Milov *et al.*, 2020 ▸), we also successfully utilized the *XCASCADE* and *XCascade3D* codes for semi-quantitative comparison with experimental data.

### 3.3. Electron ranges and cascade sizes

**Figure 4:**
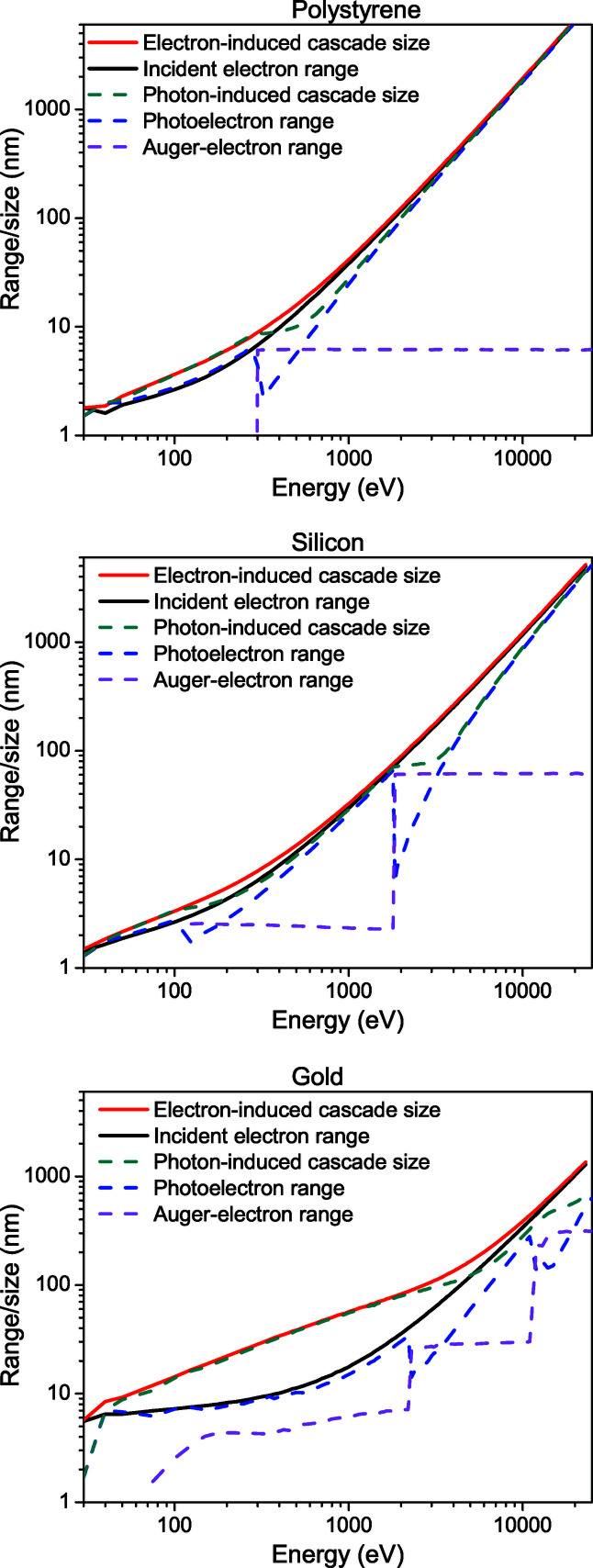
Parameters of cascades induced by an electron or a photon of given energy in polystyrene, silicon and gold. The *x* axis shows the energy of the impact particle (electron – solid curves; photon – dashed curves). The plot presents the incident electron range (depending on the incident electron energy), the photo-electron range (depending on the incident photon energy), the size of the electron-induced cascade and the size of the photon-induced cascade (see definitions in the text). Ranges of photon-induced Auger electrons are also shown.

Fig. 4 ▸ presents the parameters of photon-induced and electron-induced cascades for gold, silicon and polystyrene. This figure demonstrates several patterns. First, note that the electron-induced cascade size is typically larger than the incident electron range. This difference is especially well pronounced in heavier elements and at relatively low electron energies (*e.g.* in gold below 4000 eV). At high energies for heavy elements and at the entire energy range considered for lighter elements (*e.g.* silicon and polystyrene), the difference is small, and the electron-induced cascade size may be approximated with the incident electron range. As mentioned above, the difference is due to the fact that the incident electron on average does not travel along a straight line and its stopping point is closer to its starting point than the farthest stopping point among all the secondary electrons in the cascade.

Fig. 4 ▸ also shows that the photon-induced electron cascade is systematically smaller than the electron-induced cascade for the incident electrons of the same energy as the incident photon energy. This is expected considering the fact that an electron of a given energy needs to travel and perform a number of collisions to lose energy, whereas a photon is predominantly absorbed by the deepest shell possible (for which ionization potential is still smaller than the photon energy). Photon absorption thus creates an electron with a lower starting energy, which correspondingly travels a shorter distance before coming to a stop. Thus, photons will always produce smaller cascades than electrons of the same energy, starting from the same point in space. This effect is especially pronounced at photon energies above each ionization potential of the target. We also see in the figures that, at almost all energies considered, the photo-electron travels farther from the photo-absorption point than the Auger electrons, defining the cascade size. Only at energies in the vicinity above the ionization potentials of core shells can the situation reverse. Photo-electron ranges exhibit dips at the ionization potentials because with the excitation of each new atomic shell, the kinetic energy of the photo-electron drops, and so does the range.

**Figure 5:**
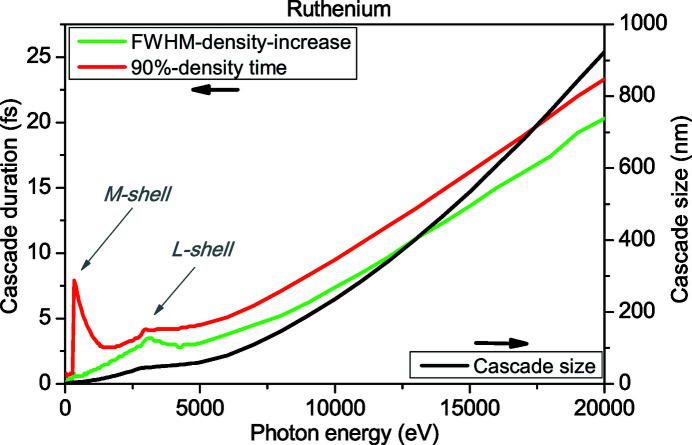
Left axis: photon-induced electron cascade duration in ruthenium defined in two ways: as the full width at half-maximum of the electron density derivative (green curve), and as the time instant where electron density reaches 90% of the total density (red curve). Right axis: photon-induced electron cascade size in ruthenium as a function of incident photon energy (black curve).

Medvedev (2015 ▸) reported that the electron-cascade duration as a function of photon energy increases over time, with characteristic dips at energies corresponding to ionization potentials of target element shells. The size of the cascades also increases with the photon energy; however, at a different rate, see Fig. 5 ▸, where different slopes of the curves are shown. This nonlinear dependence between the cascade duration and its size can be attributed to the fact that the electron scattering (elastically and inelastically) has a preferential direction to scatter forward more and more with increasing electron energy (Plante & Cucinotta, 2009 ▸). This increases the cascade range in addition to the fact that an electron simply travels further.

Also note that since the atomic approximation and approximation of the low fluence are used for the cross sections in *XCascade3D*, all the results on the electron ranges, cascade times and sizes have a linear dependence on the atomic density. The ranges and times are inversely proportional to the density. This way, for example, results presented on diamond may be rescaled for graphite or amorphous carbon without the need for a separate MC simulation. The densities of materials used in the current simulations are as follows: ρ(Au) = 19.32 g cm^−3^, ρ(B_4_C) = 2.37 g cm^−3^, ρ(diamond) = 3.52 g cm^−3^, ρ(Ni) = 8.908 g cm^−3^, ρ(polystyrene) = 1.04 g cm^−3^, ρ(Ru) = 12.3 g cm^−3^, ρ(Si) = 2.33 g cm^−3^, ρ(SiC) = 2.85 g cm^−3^, ρ(Si_3_N_4_) = 3.17 g cm^−3^ and ρ(W) = 19.3 g cm^−3^.

### 3.4. Photon-induced cascade sizes in various targets

**Figure 6:**
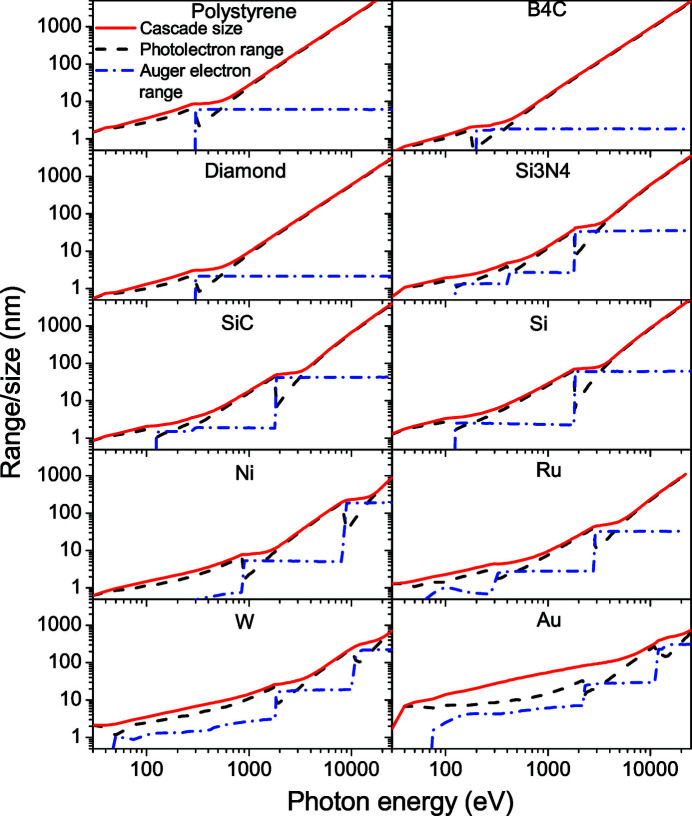
Sizes of the cascades induced by a photon of given energy in various materials, compared with the photo- and Auger-electron ranges. Targets are arranged by increase of the average atomic number.

Fig. 6 ▸ shows photon-induced cascade sizes in various materials: elemental metals (Au, Ni, Ru, W), semiconductors (Si, B_4_C, polystyrene) and insulators (diamond, SiC, Si_3_N_4_). In all of them, we can see characteristic kinks in the total cascade size when the photon energy crosses an ionization potential of a target element. At the crossings, the main contribution to the cascade size comes from the Auger electron instead of the photo-electron. Then, with further increase of the photon energy, the average Auger-electron range stays nearly constant, whereas the photo-electron range increases continually and becomes almost equal to the cascade size.

In this work, the Auger-electron range is calculated using the Monte Carlo simulation, and not within the continuous slowing-down approximation. This is why the Auger-electron ranges do not always stay constant for photon energies between ionization potentials of a target element, which is especially pronounced in heavy elements. With the change of the photo-absorption cross section, the probability of creating an Auger electron may change, which results in changes in the average Auger-electron ranges. We clearly observe this in an example of ruthenium at low energies where the Auger-electron range decreases smoothly due to increasing valence-shell photoabsorption in this photon energy region.

**Figure 7:**
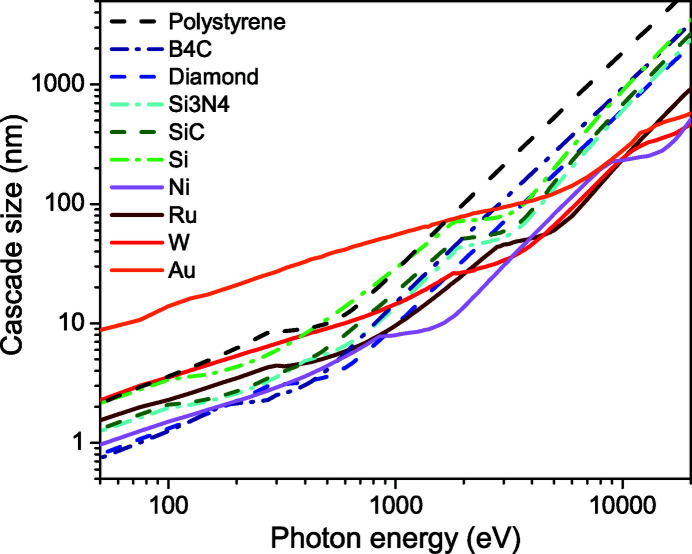
Sizes of the cascades induced by a photon of given energy in different materials. Materials are listed by increase of the average atomic number.

Fig. 7 ▸ compares photon-induced cascade sizes in the materials studied. This demonstrates that cascades in solids increase in size with the photon energy at different rates: light-element materials (polystyrene, diamond, SiC, Si_3_N_4_) with the relatively low ionization potentials of *K*-shells produce larger cascades with the increase of photon energy. The heavier-element materials (gold, ruthenium, tungsten, nickel) with deeper-lying core holes have significantly more compact cascades at high X-ray energies. This result suggests that the characteristic drops in the cascade size can be used to reduce the cascade spread by adjusting the photon energy in a given material.

Depending on the application, cascade size may thus be controlled by selecting a material with desired properties. Alternatively, for a given material, the photon energy tailored across ionization potentials may noticeably change the electron spread, thereby changing the density of excited electrons in a cascade. Since the induced electron density has practical implications [*e.g.* defining the damage threshold of the material (Ziaja *et al.*, 2015 ▸), or transient changes of the optical properties (Medvedev, 2015 ▸)], the findings presented for the X-ray-induced cascade sizes may be used in future experiments and applications.

Note that, when designing a particular experiment or application, it is important to take into account experimental conditions that directly influence the cascading process, but are not considered here, *e.g.* photon angle of incidence, particular polarization and electron escape from the irradiated target. These effects can be consistently and easily included in the framework of the *XCascade3D* model, so we expect that the model may be useful for designing specific experiments.

## 4. Conclusions

Using the Monte Carlo simulation tool *XCascade3D*, we demonstrated that the size of photo- or electron-induced electron cascades may be larger than the corresponding electron range. This effect is especially pronounced at relatively low electron energies in heavy elements, whereas at high-incident electron energy the electron range essentially coincides with the cascade size.

The simulations suggest that the cascade size induced by a photon is systematically smaller than the electron-induced cascade size in the case of equal incident energy of the electron and the photon. This effect can be attributed to the fact that photons are absorbed predominantly by the deepest possible shell (with ionization potential smaller than the photon energy), thereby producing photo- and Auger electrons with smaller energies than the incident electron energy in the case of the electron-induced cascade. Redistributing energy provided by a photon to a few electrons (photo- and Auger) shortens the cascade, making it more compact with respect to those produced by an electron impact.

We also demonstrated that targets consisting of light elements typically have larger cascades at high photon energies than those of heavy elements, owing to the fact that heavy elements have deep lying shells with ionization potential in the X-ray range. This is in addition to the fact that the photon attenuation length is also larger in heavy-element materials. The absence of deeply lying shells in light elements makes photo-electron energies higher, inducing temporally longer and spatially larger cascades.

Note that the spatial electron- and energy-density distributions produced in an electron cascade may be drastically different from uniform ones with the size estimated either in the electron range or the size of the cascade investigated here, as indicated by Follath *et al.* (2019 ▸). Whenever spatial distribution plays a role in an experiment, a dedicated study of a similar kind may be required for a quantitative description. *XCascade3D* can provide the 3D distribution of the electrons (Follath *et al.*, 2019 ▸) and their energies (Milov *et al.*, 2018 ▸, 2020 ▸) in the cascade and could therefore be useful for respective applications as well.

For practical applications where the material can be chosen freely, at a given photon energy, the cascade size may be set by choosing light-element (for larger cascades at hard X-rays) or heavy-element (for smaller cascades) targets. In applications where the material is given but the photon energy can be varied, adjusting it around the core electronic levels may shorten electron cascades. These effects may be important for the interpretation of experimental data, and for practical applications that depend on the electron densities and cascade spreads.

## Figures and Tables

**Figure 2:**
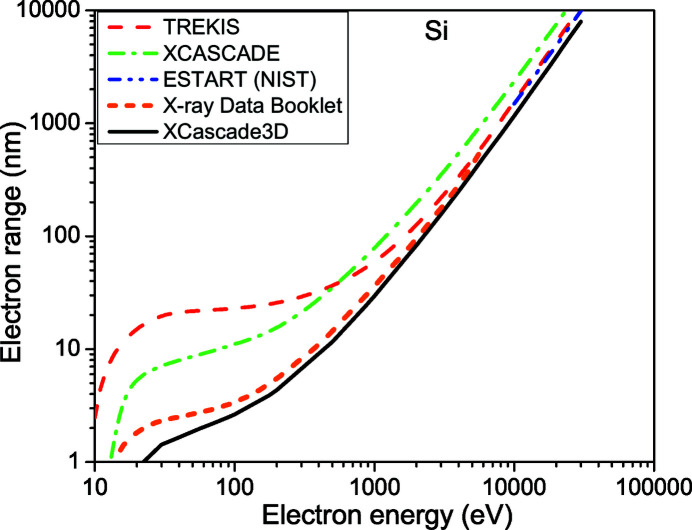
Electron range in silicon according to *XCascade3D* simulations – solid black line; *XCASCADE* estimation within the continuous-slowing-down approximation – green dash-dotted line; energy loss function integral from the NIST database – blue dash-dot-dotted line (Berger *et al.*, 1998 ▸); *TREKIS* simulation tool – red dashed line (Medvedev *et al.*, 2015 ▸); X-ray Data Booklet – orange short-dashed line (Thompson *et al.*, 2009 ▸).

**Figure 3:**
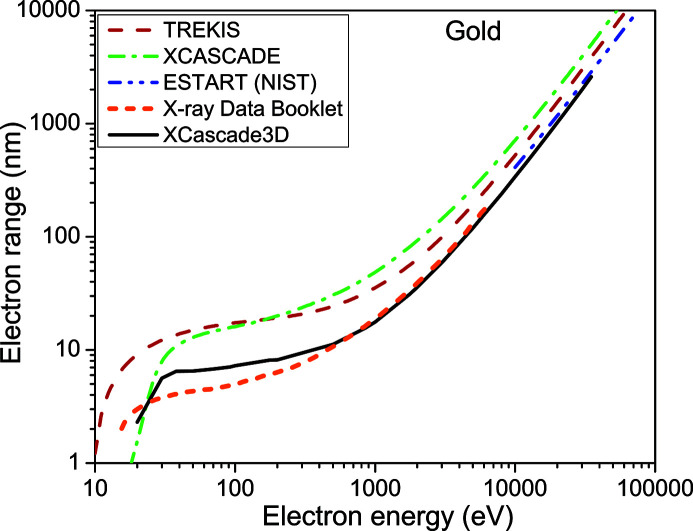
Electron range in gold according to *XCascade3D* simulations – solid black line; *XCASCADE* estimation within the continuous-slowing-down approximation – green dash-dotted line; energy loss function integral from the NIST database – blue dash-dot-dotted line (Berger *et al.*, 1998 ▸); *TREKIS* simulation tool – red dashed line (Medvedev *et al.*, 2015 ▸); X-ray Data Booklet – orange short-dashed line (Thompson *et al.*, 2009 ▸).

## References

[bb1] Ablikim, U., Bomme, C., Xiong, H., Savelyev, E., Obaid, R., Kaderiya, B., Augustin, S., Schnorr, K., Dumitriu, I., Osipov, T., Bilodeau, R., Kilcoyne, D., Kumarappan, V., Rudenko, A., Berrah, N. & Rolles, D. (2016). *Sci. Rep.* **6**, 38202. 10.1038/srep38202 PMC5133590 27910943

[bb2] Apostolova, T., Artacho, E., Cleri, F., Cotelo, M., Crespillo, M. L., Da Pieve, F., Dimitriou, V., Djurabekova, F., Duffy, D. M., García, G., García-Lechuga, M., Gu, B., Jarrin, T., Kaselouris, E., Kohanoff, J., Koundourakis, G., Koval, N., Lipp, V., Martin-Samos, L., Medvedev, N., Molina-Sánchez, A., Muñoz-Santiburcio, D., Murphy, S. T., Nordlund, K., Oliva, E., Olivares, J., Papadogiannis, N. A., Redondo-Cubero, A., Rivera de Mena, A., Sand, A. E., Sangalli, D., Siegel, J., Solov’yov, A. V., Solov’yov, I. A., Teunissen, J., Vázquez, E., Verkhovtsev, A. V., Viñals, S. & Ynsa, M. D. (2021). *Tools for Investigating Electronic Excitation: Experiment and Multi-Scale Modelling*, edited by T. Apostolova, J. Kohanoff, N. Medvedev, E. Oliva & A. Rivera. Universidad Politécnica de Madrid. Instituto de Fusión Nuclear Guillermo Velarde.

[bb3] Aquila, A., Sobierajski, R., Ozkan, C., Hájková, V., Burian, T., Chalupský, J., Juha, L., Störmer, M., Bajt, S., Klepka, M. T., Dłużewski, P., Morawiec, K., Ohashi, H., Koyama, T., Tono, K., Inubushi, Y., Yabashi, M., Sinn, H., Tschentscher, T., Mancuso, P. & Gaudin, J. (2015). *Appl. Phys. Lett.* **106**, 241905.

[bb4] Bajt, S., Prasciolu, M., Fleckenstein, H., Domaracký, M., Chapman, H. N., Morgan, A. J., Yefanov, O., Messerschmidt, M., Du, Y., Murray, K. T., Mariani, V., Kuhn, M., Aplin, S., Pande, K., Villanueva-Perez, P., Stachnik, K., Chen, J. P., Andrejczuk, A., Meents, A., Burkhardt, A., Pennicard, D., Huang, X., Yan, H., Nazaretski, E., Chu, Y. S. & Hamm, C. E. (2018). *Light Sci. Appl.* **7**, 17162. 10.1038/lsa.2017.162 PMC6060042 30839543

[bb5] Berger, M. J., Coursey, J. S., Zucker, M. A. & Chang, J. (1998). *Stopping-Power and Range Tables for Electrons, Protons, and Helium Ions.* Standard Reference Database 124. NIST, Gaithersburg, MD, USA.

[bb6] Bergmann, U., Kern, J., Schoenlein, R. W., Wernet, P., Yachandra, V. K. & Yano, J. (2021). *Nat. Rev. Phys.* **3**, 264–282. 10.1038/s42254-021-00289-3 PMC8245202 34212130

[bb7] Chalupský, J., Krzywinski, J., Juha, L., Hájková, V., Cihelka, J., Burian, T., Vyšín, L., Gaudin, J., Gleeson, A., Jurek, M., Khorsand, A. R., Klinger, D., Wabnitz, H., Sobierajski, R., Störmer, M., Tiedtke, K. & Toleikis, S. (2010). *Opt. Express*, **18**, 27836. 10.1364/OE.18.027836 21197057

[bb8] Chapman, H. N., Fromme, P., Barty, A., White, T. A., Kirian, R. A., Aquila, A., Hunter, M. S., Schulz, J., DePonte, D. P., Weierstall, U., Doak, R. B., Maia, F. R. N. C., Martin, A. V., Schlichting, I., Lomb, L., Coppola, N., Shoeman, R. L., Epp, S. W., Hartmann, R., Rolles, D., Rudenko, A., Foucar, L., Kimmel, N., Weidenspointner, G., Holl, P., Liang, M., Barthelmess, M., Caleman, C., Boutet, S., Bogan, M. J., Krzywinski, J., Bostedt, C., Bajt, S., Gumprecht, L., Rudek, B., Erk, B., Schmidt, C., Hömke, A., Reich, C., Pietschner, D., Strüder, L., Hauser, G., Gorke, H., Ullrich, J., Herrmann, S., Schaller, G., Schopper, F., Soltau, H., Kühnel, K., Messerschmidt, M., Bozek, J. D., Hau-Riege, S. P., Frank, M., Hampton, C. Y., Sierra, R. G., Starodub, D., Williams, G. J., Hajdu, J., Timneanu, N., Seibert, M. M., Andreasson, J., Rocker, A., Jönsson, O., Svenda, M., Stern, S., Nass, K., Andritschke, R., Schröter, C., Krasniqi, F., Bott, M., Schmidt, K. E., Wang, X., Grotjohann, I., Holton, J. M., Barends, T. R. M., Neutze, R., Marchesini, S., Fromme, R., Schorb, S., Rupp, D., Adolph, M., Gorkhover, T., Andersson, I., Hirsemann, H., Potdevin, G., Graafsma, H., Nilsson, B. & Spence, J. C. H. (2011). *Nature*, **470**, 73–77.

[bb9] Cullen, D. E. (2018). *A Survey of Atomic Binding Energies for use in EPICS2017*, pp. 1–60. Nuclear Data Section, International Atomic Energy Agency, Vienna, Austria.

[bb10] Dinh, T., Medvedev, N., Ishino, M., Kitamura, T., Hasegawa, N., Otobe, T., Higashiguchi, T., Sakaue, K., Washio, M., Hatano, T., Kon, A., Kubota, Y., Inubushi, Y., Owada, S., Shibuya, T., Ziaja, B. & Nishikino, M. (2019). *Commun. Phys.* **2**, 150.

[bb11] Follath, R., Koyama, T., Lipp, V., Medvedev, N., Tono, K., Ohashi, H., Patthey, L., Yabashi, M. & Ziaja, B. (2019). *Sci. Rep.* **9**, 2029. 10.1038/s41598-019-38556-0 PMC6375931 30765754

[bb12] Gaudin, J., Sinn, H., Samoylova, L., Yang, F. & Tschentscher, T. (2009). *Proc. SPIE*, **7361**, 736105.

[bb13] Harmand, M., Coffee, R., Bionta, M., Chollet, M., French, D., Zhu, D. M., Fritz, D. T., Lemke, H., Medvedev, N., Ziaja, B., Toleikis, S. & Cammarata, M. (2013). *Nat. Photon.* **7**, 215–218.

[bb14] Jacoboni, C. & Reggiani, L. (1983). *Rev. Mod. Phys.* **55**, 645–705.

[bb15] Jenkins, T. M., Nelson, W. R. & Rindi, A. (1988). Editors. *Monte Carlo Transport of Electrons and Photons.* NY: Plenum Press.

[bb16] Kastirke, G., Schöffler, M. S., Weller, M., Rist, J., Boll, R., Anders, N., Baumann, T. M., Eckart, S., Erk, B., De Fanis, A., Fehre, K., Gatton, A., Grundmann, S., Grychtol, P., Hartung, A., Hofmann, M., Ilchen, M., Janke, C., Kircher, M., Kunitski, M., Li, X., Mazza, T., Melzer, N., Montano, J., Music, V., Nalin, G., Ovcharenko, Y., Pier, A., Rennhack, N., Rivas, D. E., Dörner, R., Rolles, D., Rudenko, A., Schmidt, P., Siebert, J., Strenger, N., Trabert, D., Vela-Perez, I., Wagner, R., Weber, T., Williams, J. B., Ziolkowski, P., Schmidt, L. P. H., Czasch, A., Trinter, F., Meyer, M., Ueda, K., Demekhin, P. V. & Jahnke, T. (2020). *Phys. Rev. X*, **10**, 021052.

[bb17] Keski-Rahkonen, O. & Krause, M. O. (1974). *At. Data Nucl. Data Tables*, **14**, 139–146.

[bb18] Kim, Y.-K. & Rudd, M. (1994). *Phys. Rev. A*, **50**, 3954–3967. 10.1103/physreva.50.3954 9911367

[bb19] Koyama, T., Yumoto, H., Miura, T., Tono, K., Togashi, T., Inubushi, Y., Katayama, T., Kim, J., Matsuyama, S., Yabashi, M., Yamauchi, K. & Ohashi, H. (2016). *Rev. Sci. Instrum.* **87**, 051801. 10.1063/1.4950723 27250368

[bb20] Lipp, V., Medvedev, N. & Ziaja, B. (2017). *Proc. SPIE*, **10236**, 102360H.

[bb21] Mecseki, K., Höppner, H., Büscher, M., Tkachenko, V., Medvedev, N., Bekx, J. J., Lipp, V., Piekarz, P., Windeler, M., Tisch, J. W. G., Walke, D. J., Nakatsutsumi, M., Prandolini, M. J., Glownia, J. M., Sato, T., Sikorski, M., Chollet, M., Teubner, U., Robinson, J., Toleikis, S., Ziaja, B. & Tavella, F. (2018). *Appl. Phys. Lett.* **113**, 114102.

[bb22] Medvedev, N. (2015). *Appl. Phys. B*, **118**, 417–429.

[bb23] Medvedev, N. (2019). *Appl. Phys. B*, **125**, 80.

[bb24] Medvedev, N. A., Rymzhanov, R. A. & Volkov, A. E. (2015). *J. Phys. D Appl. Phys.* **48**, 355303.

[bb25] Medvedev, N. A., Volkov, A. E., Rethfeld, B. & Shcheblanov, N. S. (2010). *Nucl. Instrum. Methods Phys. Res. B*, **268**, 2870–2873.

[bb26] Medvedev, N., Tkachenko, V. & Ziaja, B. (2015). *Contrib. Plasma Phys.* **55**, 12–34.

[bb27] Melissinaki, V., Gill, A. A., Ortega, I., Vamvakaki, M., Ranella, A., Haycock, J. W., Fotakis, C., Farsari, M. & Claeyssens, F. (2011). *Biofabrication*, **3**, 045005. 10.1088/1758-5082/3/4/045005 21931197

[bb28] Milov, I., Lipp, V., Ilnitsky, D., Medvedev, N., Migdal, K., Zhakhovsky, V., Khokhlov, V., Petrov, Y., Inogamov, N., Semin, S., Kimel, A., Ziaja, B., Makhotkin, I. A., Louis, E. & Bijkerk, F. (2020). *Appl. Surf. Sci.* **501**, 143973.

[bb29] Milov, I., Makhotkin, I. A., Sobierajski, R., Medvedev, N., Lipp, V., Chalupský, J., Sturm, J. M., Tiedtke, K., de Vries, G., Störmer, M., Siewert, F., van de Kruijs, R., Louis, E., Jacyna, I., Jurek, M., Juha, L., Hájková, V., Vozda, V., Burian, T., Saksl, K., Faatz, B., Keitel, B., Plönjes, E., Schreiber, S., Toleikis, S., Loch, R., Hermann, M., Strobel, S., Nienhuys, H. K., Gwalt, G., Mey, T., Enkisch, H. & Bijkerk, F. (2018). *Opt. Express*, **26**, 19665–19685. 10.1364/OE.26.019665 30114137

[bb30] Nass, K. (2019). *Acta Cryst.* D**75**, 211–218. 10.1107/S2059798319000317 PMC6400258 30821709

[bb31] Okhrimovskyy, A., Bogaerts, A. & Gijbels, R. (2002). *Phys. Rev. E*, **65**, 037402. 10.1103/PhysRevE.65.037402 11909325

[bb32] Patterson, B. D. (2014). *Crystallogr. Rev.* **20**, 242–294.

[bb33] Pikuz, T., Faenov, A., Matsuoka, T., Matsuyama, S., Yamauchi, K., Ozaki, N., Albertazzi, B., Inubushi, Y., Yabashi, M., Tono, K., Sato, Y., Yumoto, H., Ohashi, H., Pikuz, S., Grum-Grzhimailo, A. N., Nishikino, M., Kawachi, T., Ishikawa, T. & Kodama, R. (2015). *Sci. Rep.* **5**, 17713. 10.1038/srep17713 PMC4669527 26634431

[bb34] Plante, I. & Cucinotta, F. (2009). *New J. Phys.* **11**, 063047.

[bb35] Riedel, R., Al-Shemmary, A., Gensch, M., Golz, T., Harmand, M., Medvedev, N., Prandolini, M. J., Sokolowski-Tinten, K., Toleikis, S., Wegner, U., Ziaja, B., Stojanovic, N. & Tavella, F. (2013). *Nat. Commun.* **4**, 1731. 10.1038/ncomms2754 23591898

[bb36] Salvat, F. & Fern, M. (2015). *PENELOPE-2014 – a Code System for Monte Carlo Simulation of Electron and Photon Transport.* Nuclear Energy Agency, Organisation for Economic Co-operation and Development.

[bb37] Spence, J. C. H. (2017). *Struct. Dyn.* **4**, 044027. 10.1063/1.4984606 PMC5453805 28653018

[bb38] Thompson, A., Vaughan, D., Kirz, J., Attwood, D., Gullikson, E., Howells, M., Kim, K.-J., Kortright, J., Lindau, I., Pianetta, P., Robinson, A., Underwood, J., Williams, G. & Winick, H. (2009). *X-ray Data Booklet*, p. 457. Center for X-ray Optics and Advanced Light Source, Lawrence Berkeley National Laboratory, Berkeley, USA.

[bb39] Thor, J. J. van & Madsen, A. (2015). *Struct. Dyn.* **2**, 014102. 10.1063/1.4906354 PMC4711627 26798786

[bb40] Tkachenko, V., Lipp, V., Büscher, M., Capotondi, F., Höppner, H., Medvedev, N., Pedersoli, E., Prandolini, M. J., Rossi, G. M., Tavella, F., Toleikis, S., Windeler, M., Ziaja, B. & Teubner, U. (2021). *Sci. Rep.* **11**, 5203. 10.1038/s41598-021-84677-w PMC7970863 33664337

[bb41] Ziaja, B., Medvedev, N., Tkachenko, V., Maltezopoulos, T. & Wurth, W. (2015). *Sci. Rep.* **5**, 18068. 10.1038/srep18068 PMC4676029 26655671

